# Factors associated with antiretroviral treatment initiation amongst HIV-positive individuals linked to care within a universal test and treat programme: early findings of the ANRS 12249 TasP trial in rural South Africa

**DOI:** 10.1080/09540121.2016.1164808

**Published:** 2016

**Authors:** Sylvie Boyer, Collins Iwuji, Andréa Gosset, Camelia Protopopescu, Nonhlanhla Okesola, Mélanie Plazy, Bruno Spire, Joanna Orne-Gliemann, Nuala McGrath, Deenan Pillay, François Dabis, Joseph Larmarange

**Affiliations:** a INSERM, UMR_S 912, « Sciences Economiques & Sociales de la Santé et Traitement de l’Information Médicale » (SESSTIM), Marseille, France; b Aix Marseille Université, UMR_S 912, IRD, Marseille, France; c Africa Centre for Population Health, University of KwaZulu-Natal, Somkhele, South Africa; d Research Department of Infection and Population Health, University College London, London, UK; e ORS PACA, Observatoire Régional de la Santé Provence-Alpes-Côte d’Azur, Marseille, France; f ISPED, Centre Inserm U1219 Bordeaux Population Health, University of Bordeaux, Bordeaux, France; g INSERM, ISPED, Centre Inserm U1219 Bordeaux Population Health, Bordeaux, France; h Faculty of Medicine and Faculty of Human, Social and Mathematical Sciences, University of Southampton, Southampton, UK; i Faculty of Medical Sciences, University College London, London, UK; j CEPED (Centre Population & Développement-UMR 196-Paris Descartes/IRD), IRD (Institut de Recherche pour le Développement), Paris, France

**Keywords:** HIV infection, universal test and treat strategy, early antiretroviral treatment, TasP trial, South Africa

## Abstract

Prompt uptake of antiretroviral treatment (ART) is essential to ensure the success of universal test and treat (UTT) strategies to prevent HIV transmission in high-prevalence settings. We describe ART initiation rates and associated factors within an ongoing UTT cluster-randomized trial in rural South Africa. HIV-positive individuals were offered immediate ART in the intervention arm vs. national guidelines recommended initiation (CD4≤350 cells/mm^3^) in the control arm. We used data collected up to July 2015 among the ART-eligible individuals linked to TasP clinics before January 2015. ART initiation rates at one (M1), three (M3) and six months (M6) from baseline visit were described by cluster and CD4 count strata (cells/mm^3^) and other eligibility criteria: ≤100; 100–200; 200–350; CD4>350 with WHO stage 3/4 or pregnancy; CD4>350 without WHO stage 3/4 or pregnancy. A Cox model accounting for covariate effect changes over time was used to assess factors associated with ART initiation. The 514 participants had a median [interquartile range] follow-up duration of 1.08 [0.69; 2.07] months until ART initiation or last visit. ART initiation rates at M1 varied substantially (36.9% in the group CD4>350 without WHO stage 3/4 or pregnancy, and 55.2–71.8% in the three groups with CD4≤350) but less at M6 (from 85.3% in the first group to 96.1–98.3% in the three other groups). Factors associated with lower ART initiation at M1 were a higher CD4 count and attending clinics with both high patient load and higher cluster HIV prevalence. After M1, having a regular partner was the only factor associated with higher likelihood of ART initiation. These findings suggest good ART uptake within a UTT setting, even among individuals with high CD4 count. However, inadequate staffing and healthcare professional practices could result in prioritizing ART initiation in patients with the lowest CD4 counts.

## Introduction

There is substantial evidence that antiretroviral treatment (ART) provides strong health benefits at both individual and population levels. ART improves morbidity and mortality, including in HIV-positive individuals with asymptomatic infection and high CD4 counts (INSIGHT START Study Group et al., 2015; TEMPRANO ANRS 12136 Study Group et al., 2015). ART at higher CD4 counts has also been shown to reduce HIV transmission to uninfected sexual partners by maintaining viral load at undetectable level (Cohen et al., 2011; Donnell et al., 2010). Furthermore, a recent study demonstrated that increased ART coverage at lower CD4 counts was associated with a significant decrease in HIV incidence at the population level (Tanser, Bärnighausen, Grapsa, Zaidi, & Newell, 2013). This inverse relationship between ART coverage and HIV incidence together with the clear clinical benefits of extended ART use are the principles underlying the adoption of the universal test and treat (UTT) strategy to prevent HIV transmission in high-prevalence settings.

However, challenges in the HIV-care cascade are well documented (Iwuji et al., 2015) with low HIV test uptake as well as poor linkage to care and delay in initiating ART amongst eligible individuals in care often cited as barriers to achieving these benefits (Fox et al., 2014; Kranzer, Govindasamy, Ford, Johnston, & Lawn, 2012; McNairy et al., 2015; Musheke et al., 2013).

In recent years, the CD4 criterion for ART initiation evolved towards earlier initiation in WHO’s recommendations and subsequent national recommendations: ≤350 cells/mm^3^ in 2010, ≤500 cells/mm^3^ in 2013, regardless of CD4 count in 2015 (WHO, 2010, 2013, 2015), based on evidence from clinical trials (TEMPRANO ANRS 12136 Study Group et al., 2015). However, there is little evidence from “real life” settings on the acceptability and uptake of early ART in mostly asymptomatic individuals in care who have a CD4 > 350 count cells/mm^3^.

The UTT TasP trial which is ongoing in rural South Africa provides the opportunity to study this issue. We aimed to describe the uptake of ART according to CD4 count strata and associated factors among HIV-positive individuals who linked to care after home-based HIV testing.

## Methods

### Study setting and design

The ANRS 12249 TasP trial is a cluster-randomized trial implemented in the Hlabisa sub-district, located in northern KwaZulu-Natal in South-Africa, which is a largely rural area with scattered homesteads and an estimated HIV prevalence of 29% (Zaidi, Grapsa, Tanser, Newell, & Bärnighausen, 2013). The protocol of the TasP trial is described in detail elsewhere (Iwuji et al., 2013; Orne-Gliemann et al., 2015). In summary, the trial was implemented in 22 geographic clusters over time: 4 clusters opened in March 2012, 6 clusters opened in January 2013 and 12 additional clusters opened in July 2014. Each cluster is composed of an average population of about 1000 residents ≥16 years old. The main hypothesis of the TasP trial is that HIV testing of all adult members of a community, followed by immediate ART initiation of all, or nearly all, HIV-infected participants regardless of immunological or clinical staging, will prevent onward transmission and reduce HIV incidence in this population. Only individuals aged 16 years or older and resident in the trial clusters are eligible for trial participation.

The UTT strategy being tested in the TasP trial has two main components. In both trial arms, rounds of home-based counselling and HIV testing are repeated every six months. All trial participants identified as HIV-infected are then referred to a TasP trial clinic located at less than 5 km from their homes. Each TasP trial clinic is staffed by one nurse and one counsellor irrespective of population size. Busier clinics are occasionally supported by additional staff depending on availability. In the control clusters, HIV-infected adults are offered ART according to South African guidelines which from study inception to December 2014 was having one of the following criteria: CD4 count ≤350 cells/mm^3^, WHO stage 3 or 4 or pregnancy (National Department of Health, Republic of South Africa, 2013). These criteria were expanded in January 2015 to include those with CD4 ≤ 500 cells/mm^3^, hepatitis B surface antigen positivity and HIV-positive partner in a serodiscordant relationship regardless of CD4 count (National Department of Health, Republic of South Africa, 2015). In the intervention clusters, ART initiation is offered regardless of CD4 count or clinical staging. HIV-care is also available in the public sector ART programme where HIV-positive individuals can receive ART according to the South African guidelines.

The trial was approved by the Biomedical Research Ethics Committee (BREC) of the University of Kwa-Zulu-Natal (BFC 104/11) and the Medicines Control Council of South Africa.

### Study population

This study used data collected up to 15 July 2015 in the first 10 clusters including both arms of the trial (5 × 2). The study population included HIV-positive individuals who linked to care before 1 January 2015 (prior to change in ART guidelines in South Africa), and were not on ART at the baseline clinic visit but eligible for ART initiation according to the trial protocol.

### Variables

The event of interest is ART initiation defined as the first time antiretroviral therapy was dispensed to the participant in a TasP clinic.

Explanatory variables used in the analysis included data obtained through face-to-face questionnaires during home-based visits as well as information collected at the baseline clinic visit (Iwuji et al., 2013; Orne-Gliemann et al., 2015). Information obtained during home visits (Table 1) were: sex, age, education, occupational status, household wealth, food insecurity, acceptability of “immediate” ART, perception of stigma towards people living with HIV and straight line distance between homesteads and clinic. Information obtained at the baseline clinic visit included: having a regular partner, HIV-status disclosure, social support, psychological distress [*Patient Health Questionnaire-4 score* (Kroenke, Spitzer, Williams, & Löwe, 2009; Löwe et al., 2010)], time between referral and baseline clinic visit, being newly diagnosed at referral, being ART-naïve and CD4 count at baseline combined with other eligibility criteria (WHO stage 3 or 4 and pregnancy for women). Clusters have been grouped to construct a three-category variable: (i) clusters A–D, opened in 2012, stratified as having lower HIV prevalence (21.7–24.5%), relatively high staff–patient ratios (2.1–3.1 per 100 patients) and fixed clinics; (ii) clusters E and F, opened in 2013, having comparable HIV prevalence (21.1–22.5%), high staff–patient ratios (2.7–5.7 per 100 patients) and mobile clinics opened during week days only; (iii) clusters G–J, opened in 2013, having higher HIV prevalence (32.5–39.8%), low staff–patient ratios (0.6–1.3 per 100 patients) and mobile clinics opened weekdays and Saturdays.

### Statistical analysis

Follow-up period was defined as the period between baseline clinic visit and ART initiation, with right-censoring at last follow-up clinic visit of each patient. The follow-up time for patients having only the baseline visit was defined as one day (Howards, Hertz-Picciotto, & Poole, 2007).

The association between the explanatory variables and ART initiation was assessed using an extension of the Cox proportional hazard (PH) model which includes an interaction with a step function that takes into account that covariate effects may change over time. Two different effects were tested: a “main effect” corresponding to the hazard ratio (HR) over the first month of follow-up, and a change in the main effect after 30 days (“effect change after one month”). The 30-day threshold was assessed by plotting the Kaplan–Meier survival curves (i.e., the cumulative probability of not being ART treated) according to the different categories of the covariates. The introduction of an interaction with time through a step function was also justified by the results of the test of PH assumption, based on the rescaled Schoenfeld residuals (Grambsch & Therneau, 1994) performed in the standard Cox model which showed that the PH assumption was violated for some covariates.

Potential explanatory variables having a *p*-value <0.25 for the main effect or for an effect change, for at least one category (Wald test) in the univariate analyses, were considered eligible for inclusion in the multivariate analysis. A backward stepwise procedure was then used to select variables in the final multivariate model with a *p*-value ≤0.05 (Wald test). A main effect after one month of follow-up was also computed for the multivariate model, corresponding to the product of the main effect and the effect change after one month. A global Chi-square test was performed to test the Cox specification using the interaction with time vs. the standard Cox specification in the final multivariate model.

Stata/SE 12.1 for Windows software (StataCorp, 2011) was used for all analyses.

## Results

### Study population

**Figure 1:**
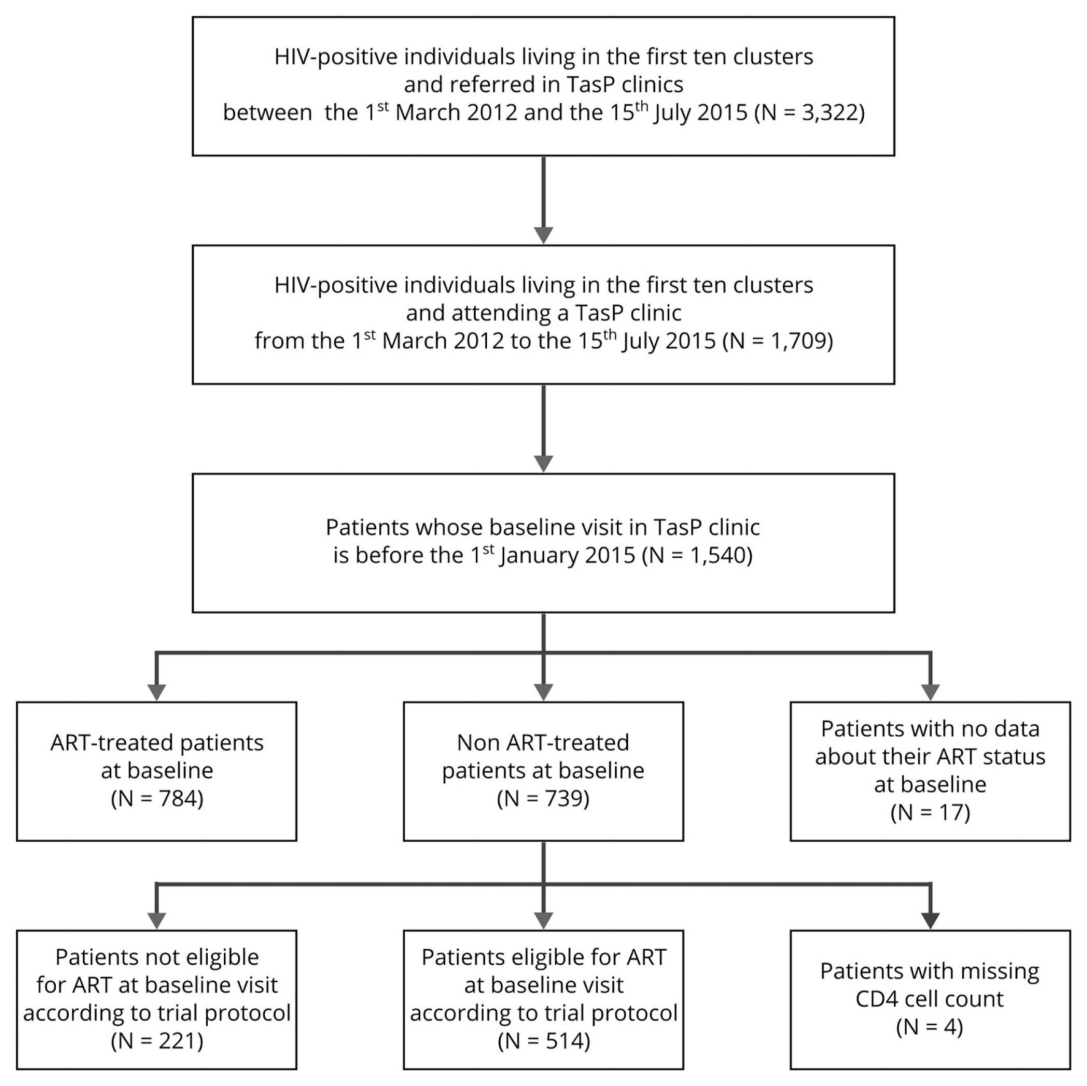
Flow chart of the study (ANRS 12249 TasP trial, rural South Africa).

The study population included 514 individuals (Figure 1). Baseline characteristics are presented in Table 1: 70.4% were women, 35.6% less than 30 years old. Most of them had a poor socio-economic status: only 22.6% completed secondary or higher education, 77.2% reported being inactive (not employed and not student) and 61.7% declared food insecurity (not having enough food for adult household members). Most reported having a regular partner (77.2%), while 15.0% had not disclosed their HIV status to anyone and nearly a quarter reported no social support (21.6%). Most of the patients (73.0%) entered into care within three months of referral. A small minority (8.8%) had less than 100 CD4/mm^3^ at baseline and 45.3% more than 350 CD4/mm^3^.

### Descriptive analysis of ART initiation

The 514 study patients had a median [interquartile range (IQR)] duration of follow-up of 1.08 [0.69; 2.07] months until ART initiation or last visit, representing 1206.4 person-months. Among them, 451 (87.7%) initiated ART during the follow-up period. Table 2 presents the rates (cumulative probabilities) of ART initiation by cluster and overall, at one month (M1), three months (M3) and six months (M6) from the baseline visit. The overall rate of ART initiation at M1 was 49.5%, increasing to 82.2% and 88.7% at M3 and M6, respectively. We also observed a large variability across clusters, especially at M1, with rates varying between 38.4% and 83.3%.

**Figure 2:**
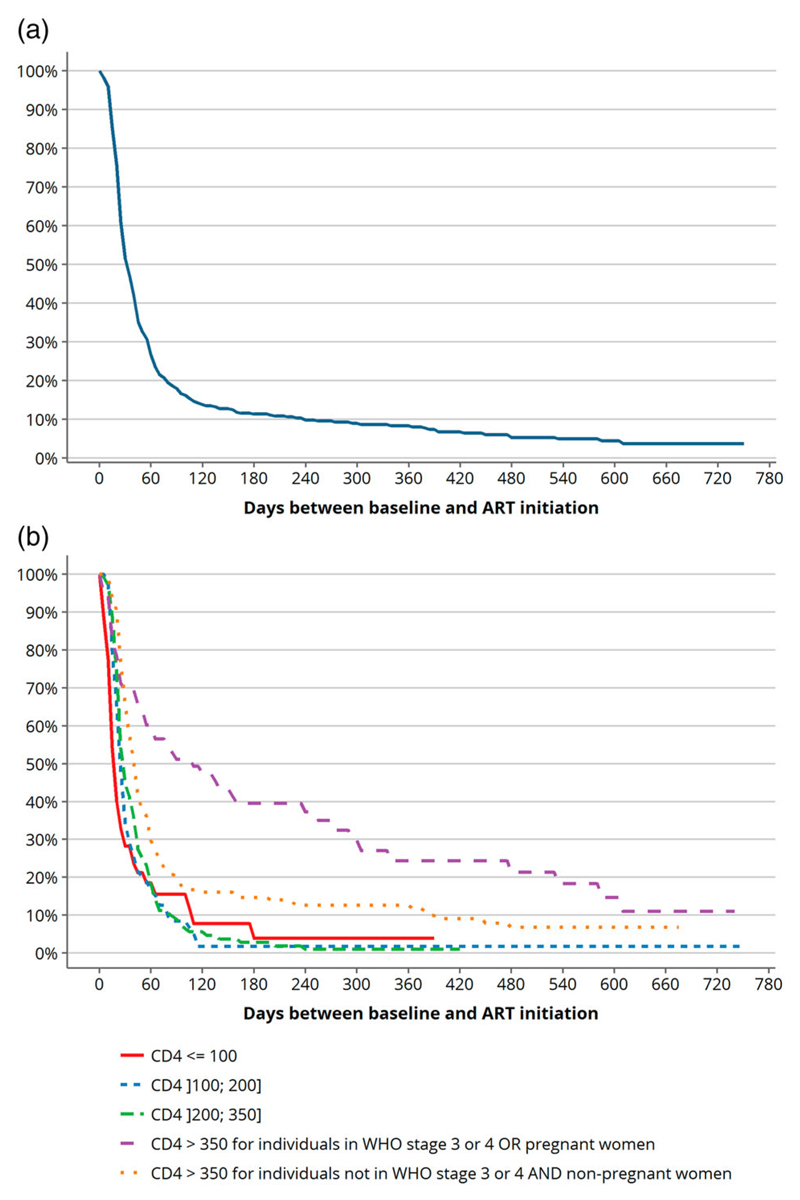
(a) Overall cumulative probability of not being ART treated among ART-eligible patients (according to trial protocol) at baseline clinic visit (Kaplan–Meier curves, ANRS 12249 TasP trial, rural South Africa, *N* = 514). (b) Cumulative probability of not being ART treated according to eligibility criteria among ART-eligible patients (according to trial protocol) at baseline clinic visit (Kaplan–Meier curves, ANRS 12249 TasP trial, rural South Africa, *N* = 514).

Figure 2 and Table 3 show, respectively, the cumulative probability of ART initiation, estimated using Kaplan–Meier curves, and the corresponding estimated rates at M1, M3 and M6 (both overall and according to CD4 count and other eligibility criteria). Rates of ART initiation at M6 were quite similar and ranged from 85.3% to 96.1% (except for the specific subgroup with CD4 ≥ 350 cells/mm^3^ and WHO stage 3/4 or pregnancy who had a rate of ART initiation of 60.5% at M6) (Table 3). However, rates of ART initiation at M1 varied substantially according to CD4 and other eligibility criteria; ranging from 55.2% to 71.8% in those with CD4 ≤ 350 cells/mm^3^ compared to 30.7% in the category WHO stage 3/4 or pregnancy and 36.9% in the category without WHO stage 3/4 or pregnancy, in those with CD4 > 350 cells/mm^3^.

### Factors associated with ART initiation

Table 1 presents factors associated with ART initiation in univariate and multivariate analyses.

Explanatory variables included in the multivariate analysis included age, educational level, household assets, food insecurity, agreeing to start ART immediately if diagnosed HIV-positive, disclosure of HIV status to partner, social support, psychological distress score, distance to clinic, CD4 count combined with WHO stage 3/4 and pregnancy for women and cluster profile.

The final multivariate model confirmed most of the statistically significant associations found in univariate analyses. First, all categories of eligible patients according to national guidelines (with CD4 between [100–200], [200–350] and CD4 > 350 for individuals in WHO stage 3/4 or pregnancy) experienced lower ART within M1 compared to those with CD4 ≤ 100 cells/mm^3^, with a gradient according to CD4 categories (main HR [95% CI]= 0.6 [0.4; 1.0], 0.5 [0.3; 0.8] and 0.2 [0.1; 0.4], respectively). This main effect was no longer statistically significant after the first month (*p* = 0.188, 0.055 and 0.098, respectively). Similarly, patients with CD4 count >350 cells/mm^3^ but without WHO stage 3/4 or pregnancy were less likely to initiate ART within M1 compared with those with CD4 count ≤100 cells/mm^3^ (HR [95% CI] = 0.3 [0.2; 0.4]) but this association was no longer significant beyond M1 (*p* = .495). Second, reporting a regular partner was not significantly associated with ART initiation within M1 in the multivariate analysis (*p* = .090), but the effect change after one month was significant, suggesting that patients without a regular partner were less likely to initiate ART after M1 (change HR [95% CI] = 0.4 [0.2; 0.7], main HR after M1 [95% CI] = 0.5 [0.4; 0.8]). Moreover, individuals in Clusters G–J were significantly less likely to initiate ART within the first month (HR [95% CI] = 0.4 [0.3; 0.6]), compared to those in Clusters A–D. However, a significant effect change after M1 (change HR [95% CI] = 1.7 [1.0; 2.9]) compensates the main effect to give a non-significant main effect after M1 (*p* = .245). Finally, despite no significant association between age and ART initiation in univariate analysis, and also no significant association over the first month of follow-up in multivariate analysis, we observed a significant effect change after M1 for the patients older than 50 years compared with those aged 16–29 years (change HR [95% CI] = 2.0 [1.1; 3.6] and main HR after M1 [95% CI] = 1.5 [1.0; 2.3]).

The result of the global Chi-square test (*p* < 10^−3^) confirmed Cox specification using the interaction with time vs. the standard Cox specification in the final multivariate model.

## Discussion

This study presents early findings on ART uptake and factors associated with ART initiation among HIV-positive individuals who had linked to care in the trial clinics and were eligible for ART at their first clinic visit within the setting of an ongoing UTT cluster randomized trial in rural KwaZulu-Natal, South Africa.

Our results show that ART uptake was high, with more than four of five individuals initiating ART within the first three months of entering care, a promising observation. Furthermore, the rates of those with CD4 > 350 cells/mm^3^ and without WHO stage 3/4 or pregnancy who initiated ART were as high as 79.8% at month 3 and 85.3% at M6. However, the concept of “immediate ART” needs to be nuanced. Even in the context of overall high ART uptake, only half the individuals initiated ART within one month with our findings suggesting a more rapid initiation in sicker patients.

Furthermore, participants’ clinical characteristics and cluster of residence were dominant in the first month amongst the factors associated with ART initiation, while the additional effects of socio-demographic variables were observed beyond the first month. Individuals with higher CD4 counts at baseline were indeed less likely to initiate ART compared to those with CD4 ≤ 100 cells/mm^3^ within the first month of entering care. However, this difference was not observed beyond the first month. Previous studies conducted at a time when ART prescription was still restricted according to clinical and immunological criteria have suggested that individuals with asymptomatic HIV disease or who do not feel sick as a result of their HIV infection are less aware of the need to initiate ART (Fox et al., 2010; Plazy, Dray-Spira, Orne-Gliemann, Dabis, & Newell, 2014). A study from the same district showed that between 2007 and 2011, only 65.5% of ART-eligible individuals initiated ART; with ART initiation being more likely in females, those ≥25 years and those with low CD4 counts (Plazy et al., 2014). However, an alternative explanation for the lack of a difference in ART initiation by CD4 count category after the first month could be that the initial difference observed was driven by healthcare professionals’ (HCPs) practices and may reflect the emergency perceived by physicians to start ART as soon as possible in individuals with CD4 count ≤100 cells/mm^3^. Conversely, for patients with CD4 count >350 cells/mm^3^, informal discussions with local HCPs suggest that more time is taken both to assess the potential risks of side effects and to prepare patients to initiate lifelong ART.

In addition, we observed a significant cluster effect on ART initiation with individuals from higher prevalence clusters who attend clinics with low staff–patient ratio being less likely to initiate ART within the first month compared to their counterparts with a higher ratio. This difference is further buttressed by the descriptive analysis showing large variations in ART initiation rates between clusters. As previously, the difference observed in ART initiation rates between clusters disappeared after the first month. This suggests that the initial delay observed was not due to patients with higher CD4 counts being reluctant to start ART but was rather due to clinics’ characteristics such as staffing capacity and workload, and potentially due to triage according to CD4 cell counts by HCP.

South Africa will likely implement soon the 2015 WHO guidelines recommending universal ART, that is, regardless of CD4 count (WHO, 2015). This will lead to large numbers of individuals in care becoming eligible for ART. It is conceivable that after many years of guidelines for ART initiation emphasizing the need to focus on HIV-positive individuals with lower CD4 counts who are at increased risk of opportunistic diseases, HCPs may continue to prioritize those with lower CD4 counts over those with higher CD4 counts. Individual benefits of ART would thus remain high [although recent clinical trials indicate clear additional benefits of early ART initiation (INSIGHT START Study Group et al., 2015; TEMPRANO ANRS 12136 Study Group et al., 2015] but the public health impact of preventing transmission may not be optimal unless the health system is strengthened to deal with the challenges of high patient flow. Innovative approaches to decongest the health system would be required such as adherence clubs, support by peers, three-monthly drug pick-ups and less frequent clinic attendance for stable patients (Duncombe et al., 2015; Ellman, 2015; Grimsrud, Lesosky, Kalombo, Bekker, & Myer, 2016; Khabala et al., 2015). Their combination will be necessary to create additional capacity in clinics to see most HIV-positive individuals initiating ART rapidly.

Regarding socio-demographic characteristics, we found that having a regular partner was associated with higher likelihood of ART initiation after one month despite no significant association during the first month. This variable may capture the psychosocial benefit of being in a stable relationship and of mutual support. This association has also been shown in adherence behaviour studies in Cameroon (Boyer et al., 2011). Furthermore, while some studies showed that women were more likely to initiate ART that men (Mugglin et al., 2012; Plazy et al., 2014), we did not find any significant association between ART initiation and gender. This result is however consistent with several recent studies conducted in Nigeria, Malawi and Uganda (Aliyu et al., 2014; Feldacker, Johnson, Hosseinipour, Phiri, & Tweya, 2012; Katz et al., 2011; MacPherson et al., 2012). In addition, two studies carried out within our trial population showed no significant association between linkage to care and gender (Plazy et al., 2015) but a significant association between HIV testing and gender with a lower acceptability of home-based HIV testing among men (Larmarange et al., 2014).

Our study has some limitations. First, our sample size may have limited the statistical power to detect some effects. Second, recent changes in South Africa regarding the adoption in January 2015 of the 2013 WHO guidelines as well as the expected adoption in the near future of the 2015 WHO guidelines may have some implications for the study’s results, analysis and interpretation. As patients with CD4 count >350 cells/mm^3^ will then become eligible for ART according to national guidelines, it is possible that the delay observed during the first month of care in ART initiation amongst individuals with higher CD4 counts may not be replicated. However, considering that large numbers of patients will become immediately eligible to initiate ART with the universal treatment guidelines, coupled with existing health-care system constraints in terms of human resources (Coovadia, Jewkes, Barron, Sanders, & McIntyre, 2009; Health Systems Trust, 2008), the delay observed in our study may be accentuated, at least at the beginning of the implementation of the new guidelines.

Our results also suggest that in the context of UTT where all HIV-positive individuals are eligible for ART, and where the concept of “pre-ART” care will gradually disappear, ART initiation could be seen as one of the last steps of a shortened entry into the care process starting with HIV diagnosis. Hence, it could be relevant to change our perspective from considering “linkage to care” and “ART initiation” as two separate processes to a global “linkage to ART” perspective, from the first “90” to the second “90” to echo UNAID’s 90–90–90 target (UNAIDS, 2014).

To conclude, our results can help inform policy on the challenges related to ART initiation in the setting of any UTT strategy. These early findings suggest a good acceptability of ART within a UTT setting, even among individuals with high CD4 counts, but revealed that ART initiation among sicker patients could remain prioritized over those with higher CD4 counts due to staffing capacity as well as HCPs’ practices that will probably take time to change.

## Supplementary Material

Appendix

## Figures and Tables

**Table 1:** Factors associated with ART initiation in patients eligible to ART (according to trial protocol) not treated at baseline clinic visit, univariate and multivariate analyses (ANRS 12249 TasP trial, rural South Africa, *N* = 514).

		Univariate analyses				Multivariate analyses					
		Main effect before 1 month		Effect change after 1 month		Main effect before 1 month		Effect change after 1 month		Main effect after 1 month	
	*N* (%) at baseline	HR [95% CI]	*p*-value	HR [95% CI]	*p*-value	aHR [95% CI]	*p*-value	aHR [95% CI]	*p*-value	aHR [95% CI]	*p*-value
**Socio-demographic and economic characteristics (home-based questionnaires)**											
*Gender*											
Male	152 (29.6)	1		1							
Female	362 (70.4)	1.0 [0.8; 1.4]	0.819	1.0 [0.7; 1.5]	0.886						
*Age (years)*											
16–29	183 (35.6)	1		1		1		1		1	
30–49	235 (45.7)	1.1 [0.8; 1.5]	0.445	1.1 [0.7; 1.7]	0.610	1.0 [0.7; 1.3]	0.967	1.3 [0.8; 2.0]	0.205	1.3 [0.9; 1.8]	0.101
+50	96 (18.7)	0.9 [0.6; 1.3]	0.448	1.4 [0.8; 2.4]	0.230	0.7 [0.5; 1.1]	0.153	2.0 [1.1; 3.6]	0.023	1.5 [1.0; 2.3]	0.074
*Educational level*											
Primary and some secondary school	398 (77.4)	1		1							
Completed secondary or higher	116 (22.6)	1.3 [1.0; 1.7]	0.069	0.7 [0.5; 1.1]	0.159						
*Having children*											
Yes	444 (86.4)	1		1							
No	57 (11.1)	1.0 [0.7; 1.5]	0.970	0.7 [0.4; 1.3]	0.289						
Missing	13 (2.5)										
*Occupational status*											
Employed	84 (16.3)	1		1							
Student	27 (5.3)	1.2 [0.6; 2.1]	0.618	0.8 [0.3; 1.9]	0.545						
Inactive (not employed and not student)	397 (77.2)	1.0 [0.7; 1.4]	0.935	0.9 [0.6; 1.5]	0.761						
Missing	6 (1.2)										
*Household wealth assets*^a^											
Low	186 (36.2)	1		1							
Middle	221 (43.0)	0.9 [0.7; 1.2]	0.372	1.2 [0.8; 1.9]	0.344						
High	104 (20.2)	0.7 [0.5; 1.1]	0.115	1.5 [0.9; 2.5]	0.119						
Missing	3 (0.6)										
*Food insecurity*^b^											
Yes	317 (61.7)	1		1							
No	183 (35.6)	1.1 [0.8; 1.4]	0.600	0.8 [0.5; 1.1]	0.183						
Missing	14 (2.7)										
*Distance from home to the closest TasP clinic within the same cluster*											
≤1 km	214 (41.6)	1		1							
>1 km	300 (58.4)	1.5 [1.1; 1.9]	0.005	0.6 [0.4; 0.9]	0.014						
**Stigma and treatment perception variables (home-based questionnaires)**											
*Thinking that people avoid HIV+ individuals*											
Agree	184 (35.8)	1		1							
Disagree + Don’t know	326 (63.4)	1.1 [0.8; 1.4]	0.476	1.0 [0.7; 1.5]	0.926						
Missing	4 (0.8)										
*Thinking that people don*’*t blame HIV+ individuals*											
Agree	265 (51.6)	1		1							
Disagree + Don’t know	247 (48.1)	0.9 [0.7; 1.1]	0.336	1.1 [0.8; 1.6]	0.551						
Missing	2 (0.4)										
*Would accept to take ART immediately if diagnosed HIV+? (Acceptability of* “*immediate*” *ART)*											
Agree	484 (94.2)	1		1							
Disagree + Don’t know	24 (4.7)	0.6 [0.3; 1.1]	0.107	1.6 [0.7; 4.0]	0.285						
Missing	6 (1.2)										
**Psychosocial variables (Clinic-based questionnaire)**											
*Having a regular partner*											
Yes	397 (77.2)	1		1		1		1		1	
No	108 (21.0)	1.3 [1.0; 1.8]	0.076	0.6 [0.4; 0.9]	0.021	1.3 [0.9; 1.8]	0.090	0.4 [0.2; 0.7]	0.001	0.5 [0.4; 0.8]	0.003
Missing	9 (1.8)										
*Disclosure of HIV status to partner*											
Yes	278 (54.1)	1		1							
No	111 (21.6)	1.3 [0.9; 1.7]	0.147	0.6 [0.4; 1.0]	0.037						
No partner	108 (21.0)	1.4 [1.0; 1.9]	0.040	0.5 [0.3; 0.8]	0.006						
Missing	17 (3.3)										
*Score of HIV disclosure (no of persons)*											
None	77 (15.0)	1		1							
One	152 (29.6)	1.0 [0.7; 1.5]	0.922	1.0 [0.6; 1.9]	0.877						
Two	115 (22.4)	0.9 [0.6; 1.3]	0.506	1.2 [0.6; 2.2]	0.634						
Three or more	166 (32.3)	0.9 [0.6; 1.4]	0.738	1.3 [0.7; 2.3]	0.437						
Missing	4 (0.8)										
*Social support*											
Yes	389 (75.7)	1		1							
No	111 (21.6)	1.1 [0.8; 1.5]	0.448	0.6 [0.4; 0.9]	0.018						
Missing	14 (2.7)										
*Psychological distress (PHQ4 score)*											
None	357 (69.5)	1		1							
Mild	132 (25.7)	1.0 [0.7; 1.3]	0.871	1.4 [0.9; 2.2]	0.091						
Moderate or severe	13 (2.5)	0.9 [0.3; 2.3]	0.775	1.3 [0.3; 4.9]	0.704						
Missing	12 (2.3)										
**Linkage to care history and clinical variables (Clinic-based questionnaire)**											
*Time between referral and baseline*											
<3 months	375 (73.0)	1		1							
≥3 months	139 (27.0)	0.9 [0.7; 1.2]	0.356	1.1 [0.7; 1.7]	0.563						
*Newly diagnosed at referral*											
No	426 (82.9)	1									
Yes	88 (17.1)	1.0 [0.7; 1.4]	0.951	0.8 [0.5; 1.4]	0.451						
*ART-naïve at baseline*											
No	85 (16.5)	1									
Yes	419 (81.5)	1.0 [0.7; 1.4]	1.0	0.8 [0.5; 1.4]	0.493						
Missing	10 (2.0)										
*Eligibility criteria and CD4 count (cells/mm^3^)*											
CD4 ≤ 100	45 (8.8)	1		1		1		1		1	
CD4 between ]100–200]	84 (16.3)	0.6 [0.4; 1.0]	0.037	2.2 [0.9; 5.3]	0.087	0.6 [0.4; 1.0]	0.055	2.7 [1.1; 6.9]	0.037	1.7 [0.8; 3.9]	0.188
CD4 between ]200–350]	152 (29.6)	0.5 [0.3; 0.7]	<10^−3^	3.6 [1.6; 8.1]	0.002	0.5 [0.3; 0.8]	0.002	4.1 [1.7; 9.7]	0.001	2.1 [1.0; 4.4]	0.055
CD4 > 350 (or missing) for individuals in WHO stage 3 or 4 OR pregnant women	59 (11.4)	0.2 [0.1; 0.4]	<10^−3^	1.9 [0.7; 5.0]	0.190	0.2 [0.1; 0.4]	<10^−3^	2.0 [0.7; 5.5]	0.175	0.5 [0.2; 1.1]	0.098
CD4 > 350 for individuals not in WHO stage 3 or 4 AND non-pregnant women^c^	174 (33.9)	0.2 [0.2; 0.4]	<10^−3^	4.0 [1.8; 9.0]	0.001	0.3 [0.2; 0.4]	<10^−3^	4.5 [1.9; 10.6]	0.001	1.3 [0.6; 2.7]	0.495
**Clusters**											
*Cluster profile*											
Clusters A–D	121 (23.5)	1		1		1		1		1	
Clusters E and F	29 (5.6)	0.5 [0.3; 1.0]	0.034	1.6 [0.7; 4.0]	0.289	0.6 [0.3; 1.1]	0.107	1.7 [0.6; 4.4]	0.292	1.0 [0.5; 2.1]	0.979
Clusters G–J	364 (70.8)	0.4 [0.3; 0.6]	<10^−3^	2.1 [1.3; 3.4]	0.002	0.4 [0.3; 0.6]	<10^−3^	1.7 [1.0; 2.9]	0.029	0.8 [0.5; 1.2]	0.245

ART, antiretroviral treatment; HR, hazard ratio; aHR, adjusted hazard ratio; CI, confidence interval

a Household wealth assets were defined in three categories using a principal component analysis on sources of energy, amenities and access to drinking water and toilet facilities (Morris, Carletto, Hoddinott, & Christiaensen, 2000).

b Food insecurity was defined as not having enough food for adult household members.

c Eligibility criteria in the intervention arm only.

**Table 2:** Cumulative probability of ART initiation, time to ART initiation and clinics staff–patient ratios by cluster (ANRS 12249 TasP trial, rural South Africa).

	Cluster A (*N* = 41)	Cluster B (*N* = 38)	Cluster C (*N* = 22)	Cluster D (*N* = 20)	Cluster E (*N* = 16)	Cluster F (*N* = 13)	Cluster G (*N* = 45)	Cluster H (*N* = 178)	Cluster I (*N* = 67)	Cluster J (*N* = 74)	Overall (*N* = 514)
**Cumulative probability^a^ [95% CI] of ART initiation and time [IQR] to ART initiation among patients eligible to ART (according to trial protocol), non-treated at baseline clinic visit (*N* = 514)**											
Cumulative probability [95% CI] of ART initiation at M1	0.641 [0.493; 0.786]	0.833 [0.696; 0.932]	0.749 [0.551; 0.909]	0.600 [0.399; 0.807]	0.437 [0.238; 0.705]	0.481 [0.250; 0.775]	0.536 [0.393; 0.692]	0.384 [0.316; 0.461]	0.475 [0.359; 0.607]	0.422 [0.317; 0.545]	0.495 [0.452; 0.540]
Cumulative probability [95% CI] of ART initiation at M3	0.949 [0.848; 0.991]	0.963 [0.848; 0.997]	0.866 [0.672; 0.973]	0.800 [0.607; 0.938]	0.922 [0.709; 0.995]	0.654 [0.398; 0.891]	0.963 [0.856; 0.996]	0.791 [0.727; 0.848]	0.674 [0.556; 0.788]	0.817 [0.719; 0.896]	0.822 [0.787; 0.855]
Cumulative probability [95% CI] of ART initiation at M6	1	0.963 [0.848; 0.997]	0.933 [0.748; 0.995]	0.900 [0.728; 0.983]	0.922 [0.709; 0.995]	0.913 [0.680; 0.995]	1	0.877 [0.820; 0.921]	0.783 [0.670; 0.878]	0.831 [0.735; 0.907]	0.887 [0.856; 0.914]
Time to ART initiation, median [IQR] (days)	23 [20; 51]	15 [14; 23]	20 [13; 28]	22 [13; 59]	29.5 [21; 34]	20 [14; 133]	27 [21; 42.5]	37 [22; 62]	28 [16; 61]	33.5 [20; 56]	28 [19; 55.5]
**Clinics staff–patient ratios^b^ (*N* = 1540)**											
Total number of patients by clinics	95	71	86	64	35	73	285	340	334	157	1540
Staff–patient ratios (per 100 patients)	2.1	2.8	2.3	3.1	5.7	2.7	0.7	0.6	0.6	1.3	NA

ART, antiretroviral treatment; M1, one month after clinic baseline visit; M3, three months after clinic baseline visit; M6, six months after clinic baseline visit; CI, confidence interval; IQR, interquartile range; NA, not-applicable.

a Cumulative probability of ART initiation is calculated over full data and evaluated at indicated times.

b Clinics staff–patient ratios are computed as the number of staff per clinic divided by the total number of patients per clinic (i.e., patients with one clinic baseline visit before 1 January 2015, *N* = 1540).

**Table 3:** Cumulative probability^a^ of ART initiation among ART-eligible patients (according to trial protocol) at baseline clinic visit, overall and according to eligibility criteria (ANRS 12249 TasP trial, rural South Africa, *N* = 514).

	*N* (%) at baseline	Cumulative probability [95% CI] of ART initiation at M1	Cumulative probability [95% CI] of ART initiation at M3	Cumulative probability [95% CI] of ART initiation at M6
*Overall*	514 (100.0)	0.495 [0.452; 0.540]	0.822 [0.787; 0.855]	0.887 [0.856; 0.914]
*Eligibility criteria and CD4 count (cells/mm^3^)*				
CD4 ≤ 100	45 (8.8)	0.718 [0.579; 0.842]	0.845 [0.717; 0.937]	0.961 [0.842; 0.997]
CD4 between ]100–200]	84 (16.3)	0.671 [0.567; 0.771]	0.916 [0.841; 0.965]	0.983 [0.923; 0.998]
CD4 between ]200–350]	152 (29.6)	0.552 [0.473; 0.635]	0.919 [0.866; 0.957]	0.972 [0.930; 0.992]
CD4 > 350 for individuals in WHO stage 3 or 4 OR pregnant women	59 (11.4)	0.307 [0.203; 0.446]	0.489 [0.367; 0.627]	0.605 [0.478; 0.735]
CD4 > 350 for individuals not in WHO stage 3 or 4 AND non-pregnant women	174 (33.9)	0.369 [0.301; 0.447]	0.798 [0.734; 0.854]	0.853 [0.795; 0.902]

ART, antiretroviral treatment; M1, one month after clinic baseline visit; M3, three months after clinic baseline visit; M6, six months after clinic baseline visit; CI, confidence interval.

a Cumulative probability of ART initiation is calculated over full data and evaluated at indicated times.
