# Supplementary material for: Factors associated with antiretroviral treatment initiation amongst HIV-positive individuals linked to care within a universal test and treat programme: early findings of the ANRS 12249 TasP trial in rural South Africa
Source: AIDS Care. Author manuscript; Available in PMC 2016 Nov 4. (PMC5096681; doi:10.1080/09540121.2016.1164808)
Supplement: Appendix [file NIHMS70183-supplement-Appendix.pdf]

Appendix: Composition of the TasP Study Group

Name	Role	Affiliation
**Investigators**		
François Dabis	Co-PI (France)	- Univ. Bordeaux, ISPED, Centre Inserm U1219, Bordeaux, France<br>- INSERM, ISPED, Centre Inserm U1219, Bordeaux, France
Deenan Pillay	Co-PI (South Africa)	- Africa Centre for Population Health, University of KwaZulu-Natal, South Africa<br>- Faculty of Medical Sciences, University College London, UK
Marie-Louise Newell	Co-PI (United Kingdom)	- Africa Centre for Population Health University of KwaZulu-Natal, South Africa<br>- Faculty of Medicine, University of Southampton, UK
**Coordinators**		
Collins Iwuji	Trial Coordinator and HIV Clinician (South Africa)	- Africa Centre for Population Health, University of KwaZulu-Natal, South Africa<br>- Research Department of Infection and Population Health, University College London, UK
Joanna Orne-Gliemann	Trial Coordinator (France)	- Univ. Bordeaux, ISPED, Centre Inserm U1219, Bordeaux, France<br>- INSERM, ISPED, Centre Inserm U1219, Bordeaux, France
**Study team**		
Till Bärnighausen	Health economics	- Africa Centre for Population Health, University of KwaZulu-Natal, South Africa<br>- Dept of Global Health & Population, Harvard School of Public Health, Harvard Univ. Boston
Eric Balestre	Epidemiology and Biostatistics	- Univ. Bordeaux, ISPED, Centre Inserm U1219, Bordeaux, France<br>- INSERM, ISPED, Centre Inserm U1219, Bordeaux, France
Sylvie Boyer	Health economics	- INSERM, UMR912 (SESSTIM), Marseille, France<br>- Aix Marseille Université, UMR_S912, IRD, Marseille, France<br>- ORS PACA, Observatoire Régional de la Santé Provence-Alpes-Côte d'Azur, Marseille, France
Alexandra Calmy	Adult Medicine	- Service des maladies infectieuses, Hôpital Universitaire de Geneve, Genève.
Vincent Calvez	Virology	- Department of virology, Hôpital Pitié-Salpétrière, Paris, France
Rosemary Dray-Spira	Social sciences	- INSERM U1018, CESP, Epidemiology of Occupational and Social Determinants of Health, Villejuif, France<br>- University of Versailles Saint-Quentin, UMRS 1018, Villejuif, France
Kobus Herbst	Data management	- Africa Centre for Population Health, University of KwaZulu-Natal, South Africa
John Imrie	Social sciences	- Africa Centre for Population Health, University of KwaZulu-Natal, South Africa<br>- Centre for Sexual Health and HIV Research, Research Department of Infection and Population, Faculty of Population Health Sciences, University College London, London, UK
Joseph Larmarange	Social sciences	- CEPED (Centre Population & Développement-UMR 196-Paris Descartes/IRD), IRD (Institut de Recherche pour le Développement), Paris, France.<br>- Africa Centre for Population Health, University of KwaZulu-Natal, South Africa

France Lert	Social Sciences	- INSERM U1018, CESP, Epidemiology of Occupational and Social Determinants of Health, Villejuif, France<br>- University of Versailles Saint-Quentin, UMRS 1018, Villejuif, France
Thembisa Makowa	Field operations	- Africa Centre for Population Health, University of KwaZulu-Natal, South Africa
Anne-Geneviève Marcelin	Virology	- Department of virology, Hôpital Pitié-Salpétrière, Paris, France
Laura March	Health economics	- INSERM, UMR912 (SESSTIM), Marseille, France<br>- Aix Marseille Université, UMR_S912, IRD, Marseille, France<br>- ORS PACA, Observatoire Régional de la Santé Provence-Alpes-Côte d'Azur, Marseille, France
Nuala McGrath	Epidemiology/Social sciences	- Academic Unit of Primary Care and Population Sciences, and Department of Social statistics and Demography, University of Southampton, United Kingdom<br>- Africa Centre for Population Health, University of KwaZulu-Natal, South Africa
Kevi Naidu	Adult medicine	- Africa Centre for Population Health, University of KwaZulu-Natal, South Africa
Nonhlanhla Okesola	Nurse manager	- Africa Centre for Population Health, University of KwaZulu-Natal, South Africa
Tulio de Oliveira	Bioinformatics	- Africa Centre for Population Health, University of KwaZulu-Natal, South Africa
Melanie Plazy	Epidemiology/social sciences	- Univ. Bordeaux, ISPED, Centre Inserm U1219, Bordeaux, France<br>- INSERM, ISPED, Centre Inserm U1219, Bordeaux, France
Tamsen Rochat	Anthropology/psychology	- HSRC, South Africa
Bruno Spire	Health economics	- INSERM, UMR912 (SESSTIM), 13006, Marseille, France<br>- Aix Marseille Université, UMR_S912, IRD, Marseille, France<br>- ORS PACA, Observatoire Régional de la Santé Provence-Alpes-Côte d'Azur, Marseille, France
Frank Tanser	Epidemiology and Biostatistics	- Africa Centre for Population Health, University of KwaZulu-Natal, South Africa
Rodolphe Thiébaut	Epidemiology and Biostatistics	- Univ. Bordeaux, ISPED, Centre Inserm U1219, Bordeaux, France<br>- INSERM, ISPED, Centre Inserm U1219, Bordeaux, France
Thembelile Zuma	Psychology/Social sciences	- Africa Centre for Population Health, University of KwaZulu-Natal, South Africa
